# Detection of ANO1 mRNA in PBMCs is a promising method for GISTs diagnosis

**DOI:** 10.1038/s41598-019-45941-2

**Published:** 2019-07-02

**Authors:** Haini Li, Ancheng Wu, Wuhui Zhu, Feng Hou, Shaoyun Cheng, Jinpeng Cao, Yufen Yan, Congxiao Zhang, Zongtao Liu

**Affiliations:** 1 Qingdao Sixth People’s Hospital, Department of Gastroenterology, Qingdao, 266001 China; 2 Qingdao University Affiliated Hospital, Department of Clinicopathology, Qingdao, 266001 China; 3 Qingdao University School of Pharmacy, Department of Pharmacology, Qingdao, 266021 China; 4 Qingdao Third People’s Hospital, Department of Clinical laboratory, Qingdao, 266021 China; 5 Qingdao Third People’s Hospital, Department of Hepatobiliary surgery, Qingdao, 266021 China

**Keywords:** Cancer genetics, Diagnostic markers

## Abstract

ANO1 is a calcium-activated chloride channel protein that has been used to diagnose GISTs after tissue biopsy. Recently, ANO1 mRNA amplification in the blood has received considerable attention as a useful method for the diagnosis of GISTs. The aim of this study was to evaluate the diagnostic ability of ANO1 mRNA in distinguishing GIST patients from healthy subjects. We constructed a logistic regression model for examining the diagnostic ability of ANO1 mRNA in comparison with conventional tumor markers, including CEA, CA199, and CA724. Our results showed that ANO1 mRNA was significantly amplified in PBMCs, the average expression level and range of ANO1 mRNA in the blood were increased along with the expression of ANO1 in the tissues, and the extent of amplification of ANO1 was associated with tumor size. In addition, ROC curve analysis showed that ANO1 mRNA in the blood had the highest specificity when compared with conventional tumor markers. Moreover, a combined analysis with ANO1 mRNA and conventional tumor markers had the highest sensitivity in diagnosing GISTs. Our study indicated that detection of ANO1 mRNA in PBMCs is a promising method for diagnosis of GISTs *in vitro*.

## Introduction

Gastrointestinal stromal tumors (GISTs) are mesenchymal tumors derived from the mesoderm that arises in the gastrointestinal tract^1,2^. Most GIST patients are asymptomatic^3,4^; however, the rate of recurrence after complete resection is as high as 33% in 5 years^5^. At present, tissue biopsy is the primary method for diagnosis of GISTs, but the inconvenience of obtaining specimens has resulted in a reduction in the screening rate. Hence, there is an urgent need to explore noninvasive methods with high sensitivity and specificity for GIST screening and diagnosis, such as liquid biopsy^6^. Clinically, serum markers for the diagnosis of digestive tract tumors are carcinoembryonic antigen (CEA)^7^, carbohydrate antigen 199 (CA199)^8^, and carbohydrate antigen 724 (CA724)^9^, which are tumor-associated antigens. CEA, CA199, and CA724 are often used to assist in the diagnosis of gastrointestinal tumors. However, none of the studies have found that tumor-associated markers in the blood have the ability to distinguish between GIST patients and healthy people.

The anoctamin-1 (ANO1) gene is localized to chromosome 11q13, a locus that is frequently amplified in a series of human cancers, such as bladder cancer, breast cancer, and head and neck squamous cell carcinoma (SCCHN)^10^. ANO1, also known as transmembrane protein 16A (TMEM16A) or gastrointestinal tumor protein 1 (DOG1), is a voltage-sensitive calcium-activated chloride channel that is frequently overexpressed in many kinds of tumors, including esophageal cancers, gastrointestinal stromal tumors, lung cancer, gastric cancer, breast cancer, prostate cancer, oral squamous cell carcinoma, pancreatic cancer, hepatocellular carcinoma and SCCHN cancer^11–21^; ANO1 has been used for GIST diagnosis in immunostained tissue specimens, even those specimens in which KIT is negative^22–24^.

Previous investigations have shown that the expression of some genes in the peripheral blood mononuclear cells (PBMCs) of breast cancer patients is correlated with the number of circulating tumor cells, tumor grade, and formation of metastases^25,26^. ANO1 mRNA has been found to be overexpressed in PBMCs of patients with GISTs, and a decline in ANO1 expression levels following surgery has been reported^27^. Our recent results showed that ANO1 mRNA expression in PBMCs is upregulated in preoperative patients with ovarian cancer and decline of ANO1 mRNA expression is noted post-operation^28^. These observations suggest that detection of ANO1 mRNA expression in PBMCs can be a diagnostic and prognostic method for cancers. However, the diagnostic capability of ANO1 mRNA in PBMCs for detection of cancer is unknown. This study aimed to explore the diagnostic role of ANO1 in detection of GISTs. Furthermore, this study aimed to evaluate the combined diagnostic efficacy of conventional biomarkers, including CEA, CA199, and CA724, and ANO1 for the diagnosis of GISTs.

In this study, we found that amplification of the ANO1 mRNA in PBMCs is correlated with tumor size of GISTs, and the average expression level and range of ANO1 mRNA in the blood are increased along with the expression of ANO1 in the tissues. Then, we constructed a logistic regression model for comparing the diagnostic ability of ANO1 mRNA with CEA, CA199, and CA724. Finally, our results indicated that ANO1 gene detection has a higher diagnostic specificity when compared with conventional biomarkers. Moreover, a combined analysis with ANO1 mRNA and conventional tumor markers can significantly increase the diagnostic ability.

## Results

### The expression of ANO1 mRNA in PBMCs is correlated with the expression of ANO1 in GIST tissues

**Figure 1:**
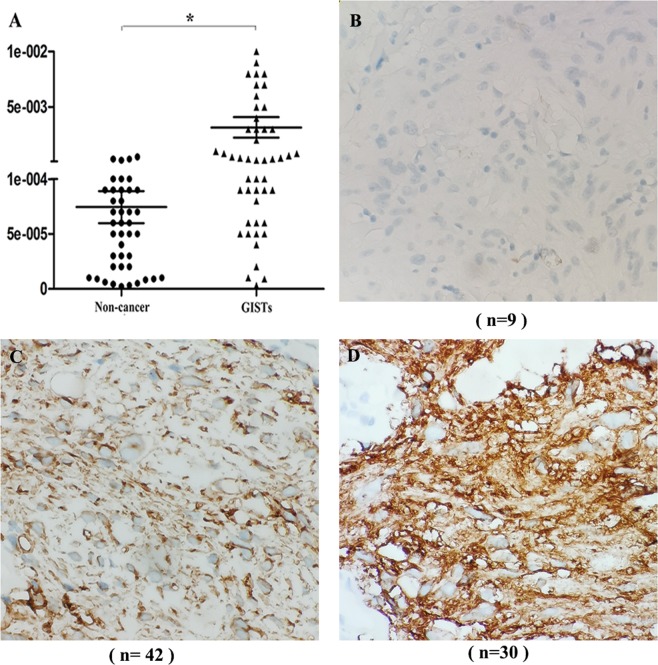
The results of ANO1 expression in PBMCs and tissues of patients with GISTs by RT-PCR and IHC assay. (**A**) expression of ANO1 mRNA in PBMCs was quantified by RT-PCR assay in 42 non-cancer individuals and 81 patients with GISTs. Compared with expression of ß-actin, ANO1 mRNA significantly upregulated in GISTs, *p < 0.05 indicates statistical significance. (**B**) Among the 81 cases of GISTs, 9 cases were negative for ANO1 expression. (**C**) Among the 81 cases of GISTs, 42 cases were low expression for ANO1. (**D**) There were 30 cases with high expression of ANO1 in 81 cases of GISTs.

To confirm the observation of upregulation of ANO1 mRNA in PBMCs from patients with GISTs, we examined the expression of ANO1 mRNA in the blood of patients with GISTs^27^. As shown in Fig. 1A, the ANO1 mRNA expression (with an average value of 3 × 10^−3^) in the GIST group relative to the expression of the housekeeping gene ß-actin was approximately 4-fold higher than the ANO1 mRNA expression (with an average value of 8.5 × 10^−4^) in the control group. Figure 1B shows the results of the 81 cases of GISTs, and it was found that 9 cases were negative for ANO1 expression. As shown in Fig. 1C, among the 81 cases of GISTs, 42 cases had low expression of ANO1. As shown in Fig. 1D, there were 30 cases with high expression of ANO1 among the 81 cases of GISTs.

To explore the correlation between the expression of ANO1 mRNA in PBMCs and the expression of ANO1 in GIST tissues, we grouped GIST tissues according to the expression of ANO1 and recorded the expression of ANO1 mRNA in PBMCs. As shown in Table 1, in the group of patients negative for ANO1 expression, the mean value of ANO1 mRNA expression in PBMCs from 9 cases was 1.0e^−5^. In the group of patients with low expression of ANO1, the mean expression of ANO1 mRNA in PBMCs from 42 cases was 3.0e^−4^. In the group of patients with high expression of ANO1, the mean value of ANO1 mRNA expression in PBMCs from 30 cases was 6.0e^−3^. The results of analysis of variance showed that the mean values of ANO1 mRNA in the three groups were statistically significant. These results showed the expression of ANO1 mRNA in PBMCs increases with an increase in the ANO1 expression in tissues; thus, suggesting *ANO1 gene* expression in PBMCs as a potential biomarker for diagnosis of GISTs.

**Table 1:** Correlation between the expression of ANO1 mRNA in PBMCs and its expression in tissues from 81 patients with GISTs.

[0 = negative]	IHC scores for ANO1 in tissues [0 = negative]		
	[1–3]	[4–6]	*p* value
No. of GISTs patients (Total = 81)	9	42	30
Expression of ANO1 mRNA (Median and range)	1.0e^−5^ (3.0e^−6^–5.0e^−4^)	3.0e^−4^ (4.0e^−5^–6.0e^−3^)	6.0e^−3^ (7.0e^−5^–4.0e^−2^) < 0.05*

Among the 81 cases of GISTs, 9 cases were negative for ANO1 expression, and the mean value of ANO1 mRNA expression in their PBMCs was 1.0e^−5^. There were 42 cases with low expression of ANO1, and the mean expression of ANO1 mRNA in their PBMCs was 3.0e^−4^. There were 30 cases with high expression of ANO1, and the mean value of ANO1 mRNA expression in their PBMCs was 6.0e^−3^. The results of analysis of variance showed that the mean values of ANO1 mRNA in the three groups were statistically significant.

*p < 0.05 indicates statistical significance.

### The upregulation of ANO1 mRNA in PBMCs is associated with clinicopathological characteristics

To compare the diagnostic efficacy of ANO1 mRNA with gastrointestinal tumor markers, we measured the levels of ANO1 mRNA, CEA, CA199, and CA724 Table 2**)**. According to the Youden index, the cutoff value for ANO1 expression that can distinguish GIST patients from healthy subjects was 1 × 10^−3^. In this study, 55 GIST patients were defined as being ANO1 positive. To investigate the correlation between ANO1 mRNA expression and pathological parameters, we have summarized the correlation between ANO1 expression and clinical features of patients with tumors in Table 3. The results showed that among these clinical correlations, ANO1 positive status was significantly correlated with tumor size, the positive rate of ANO1 expression was 81% in the group with tumor size >10 cm, the positive rate of ANO1 expression was 78% in the group with tumor size 5–10 cm, and the positive rate of ANO1 expression was 48% in the group with tumor size ≤5 cm. These results suggest that ANO1 may be involved in the development of GISTs.

**Table 2:** Measurement results of ANO1 mRNA, CEA, CA199, CA724.

Group (Number)	ANO1 mRNA (Relative value) Median (range)	CEA (ng/ml) Median (range)	CA199 (U/ml) Median (range)	CA724 (U/ml) Median (range)
GISTs (81)	3.0e^−3^ (3.0e^−6^–4.0e^−2^)	5.4 (0.8–19.3)	24.2 (0.5–145.1)	13.6 (0.5–240.2)
Non-cancer (42)	8.5e^−4^ (2.0e^−6^–5.0e^−3^)	2.4 (0.5–8.8)	7.6 (0.3–22.6)	3.4 (0.3–20.1)

**Table 3:** Correlations between expression levels of mRNA of ANO1 and clinical parameters.

	overall (n = 81)	ANO1 positive(n = 55)		ANO1 negative(n = 26)		*p* value
		N	%	N	%	
**Age**						
≤55	38	24	63	14	37	
>55	43	31	73	12	27	0.47
**Gender**						
male	40	28	70	12	30	
female	41	27	66	14	34	0.83
**Tumor location**						
Stomach	49	35	71	14	29	
Small intestine	22	13	59	9	41	
Duodenum	6	4	66	2	34	
Colorectum	4	3	75	1	25	0.7
**Histopathological**						
Epithelioid	38	29	76	9	34	
Spindle	30	18	60	12	40	
Mixed	13	8	61	5	39	0.3
**Tumor size**						
≤5 cm	29	14	48	15	52	
5–10 cm	36	28	78	8	22	*0.**018**
>10 cm	16	13	81	3	19	

ANO1 expression was significantly higher in the group with tumor size (>10 cm, positive rate, 81%) than group with tumor size (5–10 cm, positive rate, 78%) and group with tumor size (≤5 cm, positive rate, 48%). No significant correlation was found between ANO1 expression and other clinical parameters.

Values of *p < 0.05 were considered statistically significant.

### Establishment of diagnostic models and determination of diagnostic capabilities

**Figure 2:**
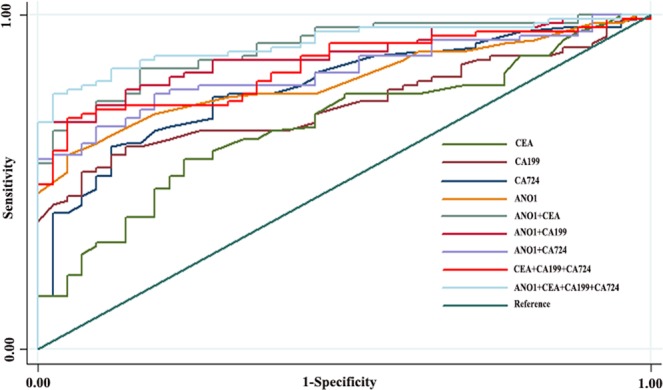
Logistic regression models for evaluating the diagnostic ability of ANO1, CEA, CA199, CA724 and jointed diagnostic ability of different marker combinations. ANO1 and CA724 had a higher AUC for diagnosis of GISTs than other tumor markers. For diagnosing GSITs, combined ANO1 with CA724, or with all of the tumor markers had better AUC.

We constructed a diagnostic model for evaluating ANO1 mRNA or tumor biomarkers to compare their diagnostic ability. As shown in Fig. 2, the area under the curve (AUC) was constructed to evaluate the diagnostic ability of ANO1, CEA, CA199, and CA724 and combined diagnostic ability of different marker combinations. Table 4 shows that the AUC, sensitivity, specificity, positive predictive value, and negative predictive value of the ANO1 mRNA model were 0.80, 64.2%, 88.1%, 91.2%, and 56%, respectively. The levels of clinical biomarkers were assessed in the same serum samples. The AUC, sensitivity, specificity, positive predictive value, and negative predictive value of CEA, CA199, and CA724 were 0.67, 69.5%, 30.6%, 71.6%, and 48.6%; 0.73, 77.8%, 40.5%, 65.8%, and 42.3%; and 0.79, 81.5%, 54.8%, 77.6%, and 60.5%, respectively. We evaluated the combined diagnostic ability of ANO1 mRNA with the clinical biomarkers, and the results were as follows: ANO1 + CEA: 0.83, 77.8%, 76.2%, 86.3%, and 64%;, ANO1 + CA199: 0.87, 81.5%, 76.2%, 86.8%, and 68.1%; ANO1 + CA724: 0.90, 83.9%, 80.8%, 89.4%, and 72.3%; CEA + CA199 + CA724: 0.84, 82.7%, 59.5%, 79.8%, and 64.1%; and ANO1 + CEA + CA199 + CA724: 0.92, 87%, 81%, 89.8%, and 77.2%, respectively. The results presented in Fig. 2 and Table 4 showed that the diagnostic ability of ANO1 and CA724 had a higher AUC, specificity, and positive predictive value for diagnosis of GISTs than the other tumor markers. For diagnosing GISTs, ANO1 combined with CA724 or with all of the tumor markers had better AUC, sensitivity, specificity, and positive predictive value.

**Table 4:** Determination of diagnostic capabilities.

	AUC	95% CI	Sensitivity	Specificity	Positive predictive value	Negative predictive value
ANO1	0.80	0.73–0.88	64.2%	88.1%	91.2%	56.0%
CEA	0.67	0.71–0.87	69.5%	30.6%	71.6%	48.6%
CA199	0.73	0.65–0.82	77.8%	40.5%	65.8%	42.3%
CA724	0.79	0.58–0.77	81.5%	54.8%	77.6%	60.5%
ANO1 + CEA	0.83	0.76–0.90	77.8%	76.2%	86.3%	64.0%
ANO1 + CA199	0.87	0.82–0.94	81.5%	76.2%	86.8%	68.1%
ANO1 + CA724	0.90	0.85–0.95	83.9%	80.8%	89.4%	72.3%
CEA + CA199 + CA724	0.84	0.77–0.91	82.7%	59.5%	79.8%	64.1%
ANO1 + CEA + CA199 + CA724	0.92	0.87–0.96	87%	81%	89.8%	77.2%

Summarized the AUC, sensitivity, specificity, positive predictive value and negative predictive value of ANO1 mRNA, CEA, CA199 and CA724 for the diagnosis of GISTs.

## Discussion

The purpose of this study was to investigate whether ANO1 can be used as a hematological marker for the diagnosis of GISTs and to evaluate the diagnostic efficacy of ANO1. In this study, we found that ANO1 mRNA was amplified in PBMCs of GIST patients and ANO1 expression was associated with tumor size. The average expression level and range of ANO1 mRNA in the blood were increased along with the expression of ANO1 in the tissues. ROC results showed that the diagnostic model using ANO1 has higher specificity in distinguishing GIST patients from healthy individuals than models using conventional biomarkers. Combined use of ANO1 and CEA, CA724, and CA199 can improve the sensitivity for diagnosis of GISTs. These results indicate that the detection of ANO1 in PBMCs could be a promising technique for diagnosis of GISTs, and ANO1 may be an effective drug target for GIST therapy.

We found that ANO1 was overexpressed in the blood, and the expression trend was consistent with the expression trend in tissues. This result suggests ANO1 gene expression in PBMCs as a potential biomarker for the diagnosis of GISTs. However, ANO1 is often overexpressed in the cell membrane or cytoplasm when cancer develops. It is unclear why the ANO1 gene is amplified in PBMCs in the blood. Studies have shown that the ANO1 gene is located in the 11q13 region of chromosomes, and this region is closely related to tumorigenesis. ANO1 may be highly expressed along with amplification of the *11q13* region, and ANO1 may also form a functional complex with molecules such as EGF receptor (EGFR) to initiate amplification of ANO1^11^. Tumor microenvironment may be one of the causes of ANO1 mRNA amplification in PBMCs of GIST patients. Recent reports have shown that tumor-infiltrating lymphocytes (TILs), including natural killer (NK) cells, monocytes, and T and B lymphocytes, are involved in an inflammatory-like tumor microenvironment and are recruited at the tumor site^25,29^. Complex tumor microenvironments and interaction of cytokines secreted by cancer cells and TILs may contribute to amplification of the ANO1 gene; however, these mechanisms require further research.

Our result of the mean level of ANO1 mRNA (3 × 10^−3^) is similar to that in a previous study, and we selected the cutoff value by using a more rigorous and scientific statistical method^27^. In our analysis of correlations between the mRNA expression level of ANO1 and clinical parameters, we found that ANO1 expression is associated with tumor size; the larger the tumor, the higher the positive rate of ANO1 expression. This result is consistent with the findings of previous studies; thus, indicating that ANO1 may play an important role in tumorigenesis of GISTs^27,30^. Although study^31^ considered tumor site is an independent prognostic factors for GISTs recurrence, while our results showed that ANO1 mRNA expression is not associated with the tumor location and pathological type is consistent with previous studies^6^, this showed that no difference may be caused by small samples, so more samples are needed to research. Further study should be performed to test the change of *ANO1 gene* expression in PBMCs from patients before and after surgery.

ANO1 is found to be widely expressed in GISTs, and it has been used as a specific immunohistochemical marker for GISTs^22,27^. Although tissue biopsy for c-KIT or ANO1 is an effective way to diagnose GISTs, patients are less receptive towards this method because of the inconvenience and invasiveness of this method. Serum biomarkers can be easily obtained and are non-invasive; however, a satisfactory blood biomarker with high sensitivity and specificity for the diagnosis of GISTs is currently unavailable^32^. Clinical studies have shown that gastrointestinal tumors not only cause elevated levels of CEA, CA199, and CA724, but they also promote the development of breast cancer, lung cancer, and other malignancies. Therefore, they cannot be used as specific indicators for the diagnosis of a malignant tumor^33–35^.

In this study, detection of ANO1 mRNA had the highest specificity (88.1%) and the highest positive predictive value (91.2%) but the lowest sensitivity in distinguishing GIST samples from healthy samples as compared with CEA, CA199, and CA724. High specificity indicates that the rate of misdiagnosis is low, and therefore, it can be used for diagnosis of the disease; however, low sensitivity indicates that the rate of missed diagnosis is high. If ANO1 mRNA is to be used for disease screening, sensitivity must be improved by combined use of markers^36^. Moreover, our results also found that combined analysis of ANO1 and a biomarker such as CEA, CA199, or CA724 had an even higher diagnostic ability, and a combined analysis of ANO1 and all of the biomarkers showed the highest AUC (0.92) and sensitivity (87%). These results indicate that detection of ANO1 mRNA in PBMCs may serve as a diagnostic biomarker of GISTs. Although the sensitivity of ANO1 measurement was not satisfactory, we were able to improve the screening ability of the model by using a combined analysis of several biomarkers. However, an inadequate sample size may have led to bias in statistical results; hence, more samples are needed to validate these results in a subsequent study.

In conclusion, our results indicate that ANO1 mRNA is a potential tumor marker for non-invasive diagnosis of GISTs. ANO1 mRNA detection by RT-PCR is a feasible and promising method for the diagnosis and prognosis of GISTs, which can improve the diagnostic capabilities and contribute to treatment decisions. Furthermore, ANO1 may be a potential drug target for GISTs.

## Methords

### Specimens and patients

Eighty-one samples from GIST patients and 42 samples from healthy candidates (as the control group) were obtained from the Affiliated Hospital of Qingdao University between February 2016 and October 2018. Informed consent was obtained from all of the patients included in this study, and this study was approved by the Ethics Committees of the Affiliated Hospital of Qingdao University, and the Six and the Third People’s Hospitals. All of the experiments were performed in accordance with relevant guidelines of a protocol approved by the Institutional Review Board from the Affiliated Hospital of Qingdao University. All of the patients had not received cancer treatment prior to diagnosis by histopathological examination. Exclusion criteria were as follows: presence of other benign and malignant tumors and dysfunction of important organs, pregnancy or lactation period, and simultaneous enrollment in other clinical trials.

### Immunohistochemical (IHC) staining of GIST tissue specimens

Experimental steps were performed as described in our previous research^[29]^. Two independent pathologists evaluated IHC staining of all of the tissue sections. Staining intensity of the ANO1 protein primarily localized in the cytomembrane was graded on the 0–3 scale as follows: 0 (absence of staining), 1 (weak staining), 2 (moderate staining), and 3 (strong staining). The percentage of ANO1 positive tumor cells was scored on the 0–3 scale as follows: 0 (absence of positive cells), 1 (less than 33% positive cells), 2 (33–66% positive cells), and 3 (more than 66% positive cells). The ANO1 staining score was calculated according to the staining intensity plus the percentage, which sums up to 0 to 6, where 0 represents no expression, 1–3 indicates low expression of ANO1, and 4–6 indicates high expression.

### RNA isolation, reverse transcription (RT), and quantitative PCR (qPCR)

After discarding the first 2 ml of blood, approximately 10 ml of blood was collected in EDTA vacuum tubes. The PBMCs were separated through density gradient centrifugation using Lymphocyte Separation Medium (Tianjin, China). Total RNA was extracted using TRIzol reagent (Invitrogen, USA). ANO1 complementary DNA was generated according to the Primer Script TMRT Reagent Kit (TaKaRa, Japan) protocol. Extracted cDNA was used as a template for amplification and quantification of ANO1 mRNA by real-time PCR (CFX96, Bio-Rad) using SYBR Premix Ex TaqTM II (TaKaRa, Japan). ß-actin was co-amplified for semi-quantitative comparison. Primer synthesis was performed by Sangon company (Shanghai), and primer sequences were as follows:

ANO1 forward primer: GAGCCAAAGACATCGGAATCTG;

ANO1 reverse primer: TGAAGGAGATCACACGAAGGCAT;

ß-actin forward primer: TGTTACCAACTGGGACGAC;

ß-actin reverse primer: GGTGTTGAAGGTCTCAAACAT.

CT values > 40 were considered to indicate negative results. Relative expression of the target ANO1 was calculated by using the 2^−ΔCT^ method. The reactions were performed in duplicate or triplicate.

### Serum biomarker detection

Serum levels of CEA, CA199, CA125, and CA724 were measured using the Roche Cobas 6000 system (Roche, Switzerland).

### Statistical analysis

All of the statistical analyses were performed using Stata 12.0 software. Differences in ANO1 expression between two or more groups were evaluated using a *two-tailed t test* or *variance analysis*. A logistic regression model utilizing ANO1 or conventional tumor markers was constructed for the diagnosis of GISTs. Receiver operating characteristic (ROC) curve was constructed to evaluate the diagnostic ability of ANO1 or biomarkers. Values of *p* < 0.05 were considered to be statistically significant.

## Data Availability

All authors agreed to make materials, data and associated protocols available to readers without undue qualifications in material transfer agreements.
